# A Comparative Study Between Hybrid Abutments and Standard Abutments in Implant-Supported Prosthesis: A Split-Mouth Clinical Trial

**DOI:** 10.7759/cureus.31993

**Published:** 2022-11-28

**Authors:** Mohammad-Naeem Alsaadi, Mhd. Luai Morad, Khaldoun Darwich, Shaza Kanout, Hassan A Husein

**Affiliations:** 1 Department of Fixed Prosthodontics, Faculty of Dentistry, Damascus University, Damascus, SYR; 2 Department of Oral and Maxillofacial Surgery, Faculty of Dentistry, Damascus University, Damascus, SYR

**Keywords:** implant prosthesis, hybrid abutment, titanium abutment, standard abutment, dental implants

## Abstract

Background

Implant-supported prostheses are widely used to replace extracted teeth. Therefore, studies on abutments’ designs, shapes, and benefits had increased in recent years, as the design of the standard abutment still poses many problems in periodontal and cosmetic aspects. So, could the hybrid abutment solve some of these problems?

Aim

We aim to conduct a clinical comparison between standard and hybrid abutments in terms of the state of peri-implant gingival tissues and patients’ aesthetic and functional satisfaction after the cementation of the final prostheses.

Material and methods

The study sample consisted of 10 patients, with 20 dental implants. Each patient received two implants as a standard abutment was placed over one implant and a hybrid abutment was placed over the other. Clinical assessment of the peri-implant gingival tissue and patients’ aesthetic and functional satisfaction was performed (immediately, three months, six months, and one year) after the cementation of the final prostheses. The Mann-Whitney U test was used to detect statistically significant differences between groups.

Results

The percentage of the thick gingival biotype was 80%, and the percentage of the thin gingival biotype was 20% in each group during the follow-up periods. In addition, all papilla fill the whole interdental space in all samples of the two groups after six months and one year. Finally, there were no significant differences in patients’ aesthetic satisfaction between groups during one year of follow-up (P = 0.631), and there were no significant differences in patients’ functional satisfaction between groups during one year of follow-up (P = 0.684).

Conclusion

Within the limitations of the current work, there are no differences between standard and hybrid abutments in terms of affecting the peri-implant gingival tissue and patients’ aesthetic and functional satisfaction.

## Introduction

Implant-supported prostheses are widely used to replace extracted teeth, as it provides a wide range of treatment options and could be fixed or removable. Implant-supported fixed prostheses consist of an implant body, abutment, abutment screw, and final prosthesis [1].

Abutments are defined as the extended part through the peri-implant gingival tissues, which are seated over the implant body. Two types of abutments are used during the treatment period, healing abutment and final abutment, which act as a connection between the implant and the final prosthesis [2].

The abutment morphology traditionally mimics a prepared natural tooth, with an axial wall convergence angle ranging from 6 to 20 degrees, depending on the different authors’ preferences [3]. The intrasulcular part of the abutment emerges from the implant body, expanding coronally to reach the buccolingual and mesiodistal dimensions of the prosthetic tooth. The emergence profile of the abutment associated with the emergence profile of the crown, as a defined finish line (usually a chamfer), is placed on the abutment and generally positioned subgingival on the buccal (“aesthetic”) side. Therefore, the buccal gingival contour is shaped by the abutment profile. Because of the different implant systems, appropriate abutments must be chosen to enable the prosthesis to emerge as a natural tooth [4].

Standard abutments have several advantages, including low cost, appropriate engagement in the implant body [5], and the possibility of preparing it inside or outside the mouth. However, it also has some disadvantages such as consuming longer time to prepare, especially in implants with improper placement, as it does not follow the anatomical details of the teeth’s gingival contour [6]. Final prostheses may have poor stability resulting from over-preparation in order to obtain good angulation of the abutment, and even angled abutments may not give us the required angle.

Hybrid abutments consist of a titanium metal base that engages in the implant with a lithium disilicate ceramic cover to hide the metal. Titanium is the best metal to be used in the area of ​​contact between the abutment and the implant, as the fit of the inner cone of the abutment with the inner cone of the implant ensures minimal leakage, thus preserving the peri-implant gingival tissues [7]. The ceramic section of the hybrid abutment has an unlimited variety of shapes, which gives it biological properties such as supporting the gingival tissues and giving a suitable emergency profile, in addition to its mechanical properties such as making the occlusal forces parallel to the longitudinal axis of the dental implants [8].

The aim of this study was to compare standard and hybrid abutments in terms of the state of peri-implant gingival tissues and patients’ aesthetic and functional satisfaction, after the cementation of the final prostheses.

## Materials and methods

Study design and sample size calculation

This study was a split-mouth clinical trial conducted at the Department of Fixed Prosthodontics, Faculty of Dentistry, Damascus University, Damascus, Syria. It was ethically approved by the Local Research Ethics Committee of the Faculty of Dentistry, Damascus University (UDDS-5455-02092020/SRC-5791).

The sample size was calculated using the G-Power software version 3.1.9.4 (Heinrich Heine University Düsseldorf, Düsseldorf, Germany) with a significance level of 0.05 and a power of 80%. The calculation revealed that a sample size of 10 patients was required for each group.

Patient recruitment and follow-up

Implants were performed for patients attending the Department of Fixed Prosthodontics of Damascus University Dental School, who required two adjacent or symmetrical implants (single posterior prostheses) on each side of the jaw, whether upper or lower, as each patient received one standard abutment and one hybrid abutment. Randomization was performed using a computer-generated random list (Microsoft Excel 2007, Microsoft Corporation, Redmond, WA, USA) with an allocation ratio of 1:1.

The inclusion criteria were as follows: (1) patients who need two symmetrical or adjacent posterior implants, (2) age 18-65 years, (3) absence of a topical contraindication or any treatment inconsistent with the treatment plan, (4) the patient has not been exposed to previous radiological or chemical treatment, (5) absence of systemic diseases that affect the healing of surrounding tissues such as diabetes, (6) adequate bone thickness, i.e., no need for surgical preparation or bone grafting before implantation, (7) good oral hygiene, and (8) not a smoker.

The exclusion criteria were as follows: (1) presence of nonfunctional habits such as stridor, (2) acute periodontitis, (3) previous loss of implants, (4) poor general health conditions, (5) previous radiotherapy in the head and neck area, (6) mental incompetence, and (7) orthodontic treatment.

We performed an examination for each patient, including the patient’s medical history and medications, with intraoral examination for periodontal tissues, oral hygiene, teeth adjacent to the implant area, and area of ​​loss. Appropriate radiographs (cone-beam computed tomography (CBCT) and panoramic and periapical radiography), gypsum casts, photographs, and measurements of mucosal thickness were used. Upon the completion of the case study, the appropriate treatment plan was developed, and patient consent was obtained.

Implantation procedure

The first surgical stage included inserting the implants (AnyRidge®, MegaGen, Seoul, South Korea) after conducting a radiological study using cone-beam computed tomography (CBCT) imaging. Implants were placed exactly at the level of the crestal bone with a torque of insertion (35 Newton (N)/cm).

**Figure 1:**
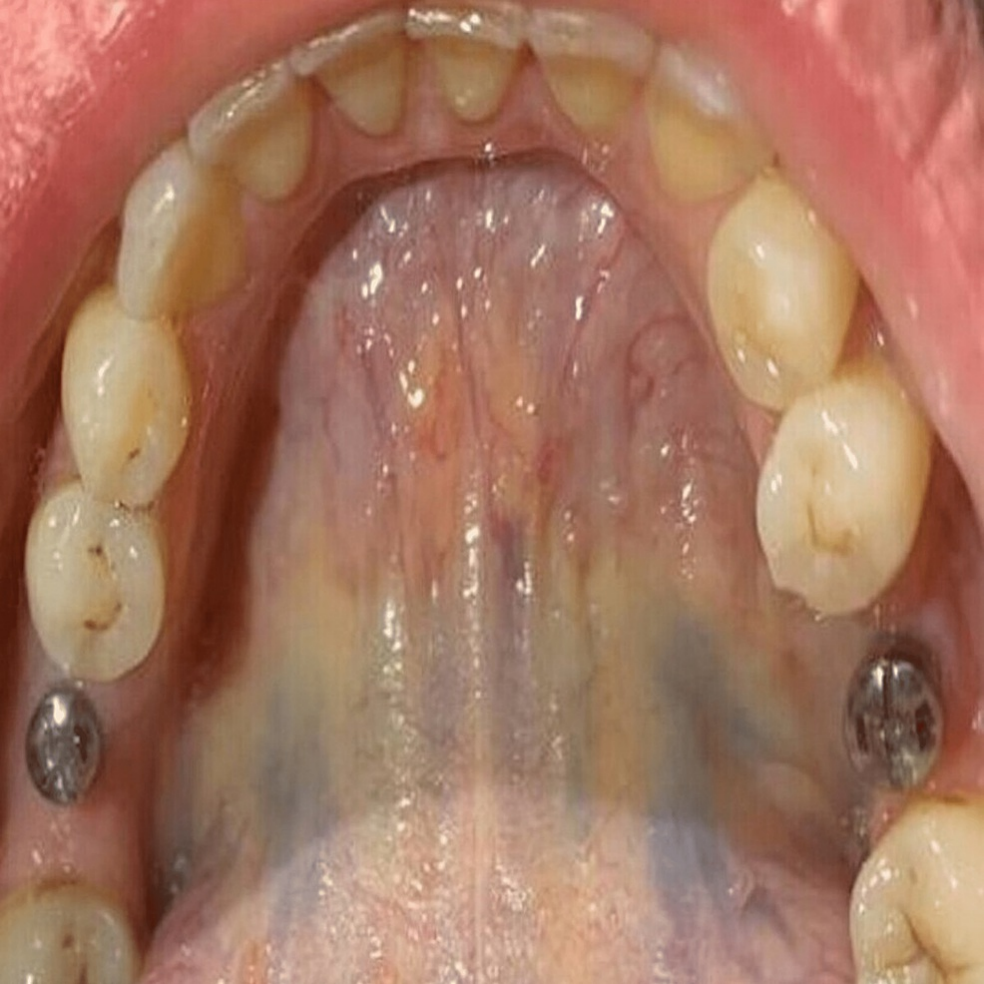
Placement of the gingival healing abutment

**Figure 2:**
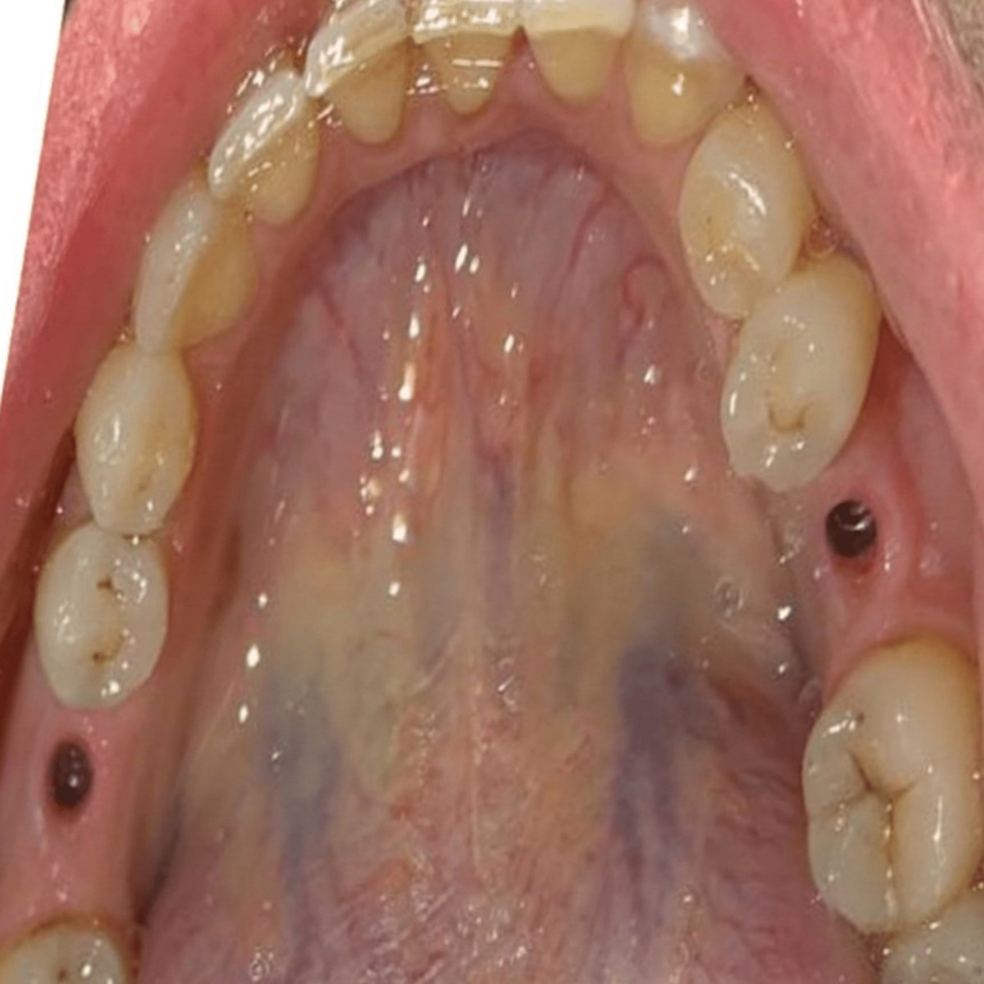
Emergence profile of the gingiva after using the healing abutments

We moved to the second surgical stage four months after the end of the first stage, which included the detection of implant position and the placement of the gingival healing abutment (Figure 1) to obtain the appropriate emergency profile (Figure 2).

Prosthetic stage

The impression was taken for the implants by adding silicone (PRESIDENT The Original, Coltene, Switzerland) in the closed tray technique using the appropriate transfer and an alternative to the laboratory implant. The gingival mask was injected similarly to placing the gingiva inside the mouth. The impression was poured using phosphate-bonded gypsum powder (Maruvest Speed, Megadental, Büdingen, Germany).

**Figure 3:**
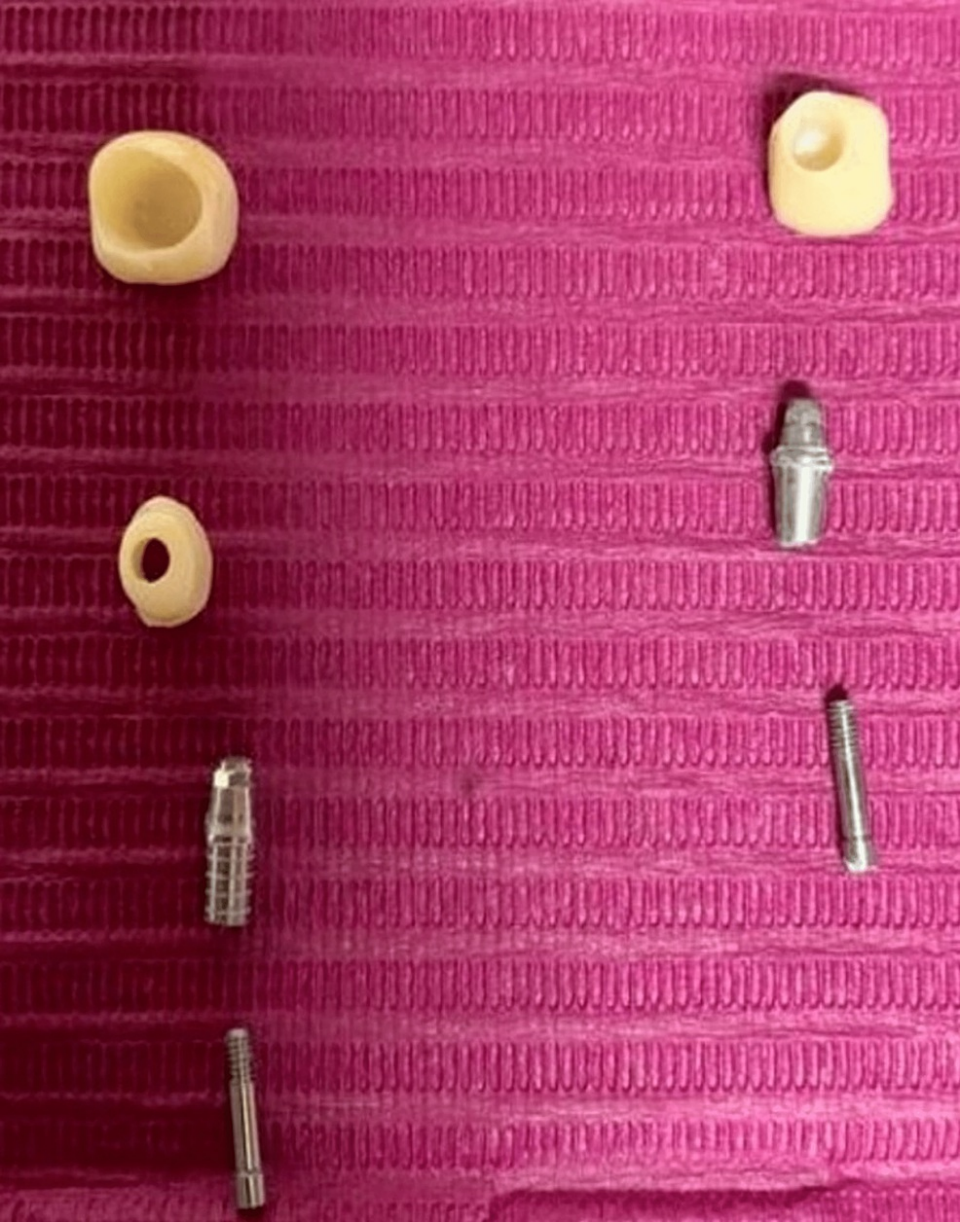
Standard and hybrid abutments’ parts with the final prostheses before fixation

**Figure 4:**
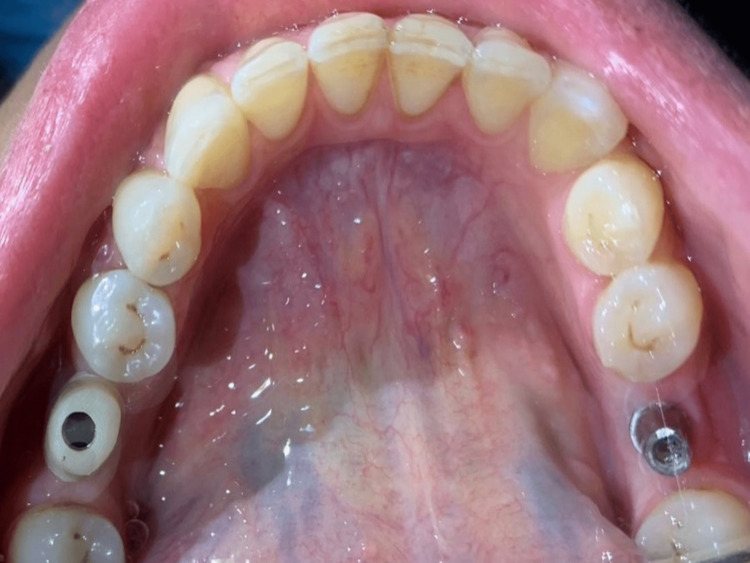
Fixing both abutments in the mouth

Abutments were selected (Figure 3) and fixed (Figure 4) to make the appropriate adjustments in terms of length and angulation to become ready to receive the computer-aided design-computer-aided manufacturing (CAD-CAM) zirconia crown.

Final prosthesis try-in

In group 1 (standard abutment), the internal fit was tested using a light silicon. Then, occlusal interference was tested in the central occlusal and lateral movements.

In group 2 (hybrid abutment), the ceramic cover was placed over the titanium abutment, tried in, and fixed with permanent adhesive cement (GC Gold Label I/Fuji I, GC India Dental Private Limited, Telangana, India). Then, the final crown was tested in the same stages as the standard abutment’s final crown.

**Figure 5:**
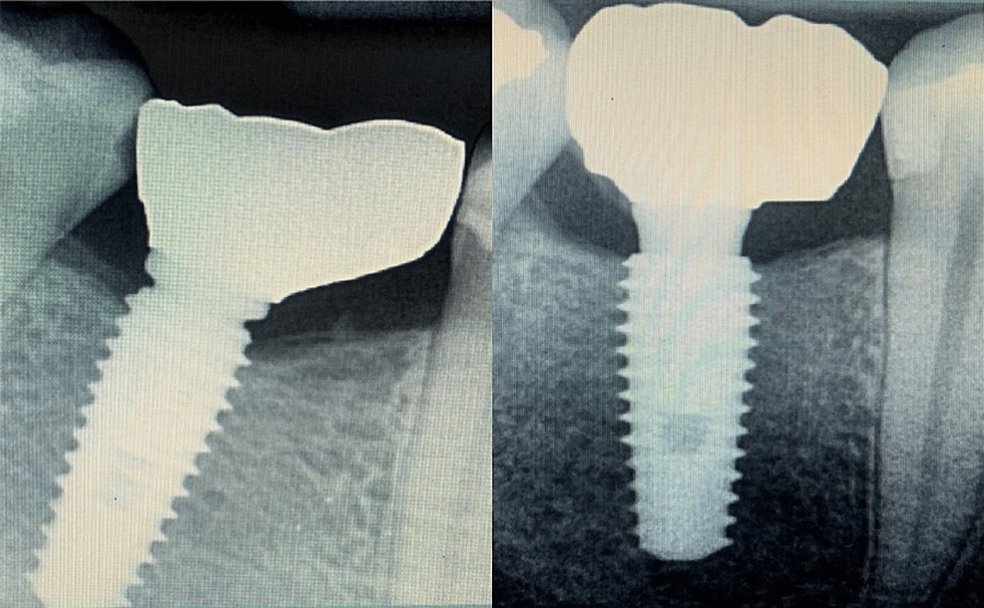
Radiographic evaluation of the final prostheses’ fitting over the implants

Finally, the final crowns were cemented using a temporary, long-term, implant-specific cement (Meta NETC, Meta Biomed, New Delhi, India), and then, post-cementation peri-apical radiography was done to ensure their correct position (Figure 5).

**Figure 6:**
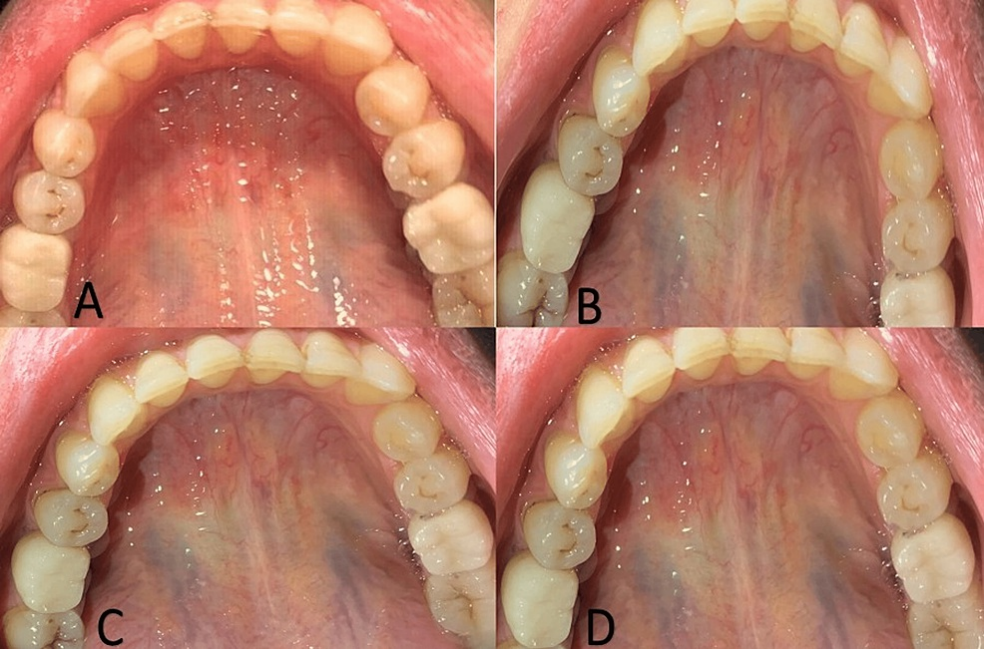
Clinical assessment of the peri-implant soft tissues at four assessment times The clinical appearance of the peri-implant soft tissues (A) immediately following the fixation of the final prosthesis, (B) after three months, (C) after six months, and (D) after one year.

Clinical assessment was done immediately following fixation and after three, six, and 12 months (Figure 6A-6D).

Outcome measures

Gingival Biotype

A periodontal probe was placed in the pocket, and the gingiva’s translucency was monitored. If a gray color appears, the gingiva is considered to be of the thin biotype and scores 0; however, if it does not appear, it is considered to be of the thick biotype and scores 1 in the appropriate field in the form for each patient.

Interdental Papilla Filling

Photographs were taken for documentation and comparison with the adjacent natural tooth. The Pink Esthetic Score (PES) was used to assess the interdental papilla filling in the distal and mesial areas [9] for each implant separately. Numbers 0, 1, and 2 were given for this evaluation as follows: 0 = the papilla is absent, 1 = the papilla does not fill its place completely, and 2 = the papilla fills its place completely (Table 1).

**Table 1:** Pink Esthetic Score The Pink Esthetic Score evaluates the aesthetic outcome of soft tissue around implant-supported single crowns by awarding seven points for the mesial and distal papilla, soft tissue level, soft tissue contour, soft tissue color, soft tissue texture, and alveolar process deficiency. Source: Fürhauser R, Florescu D, Benesch T, et al.: Evaluation of soft tissue around single‐tooth implant crowns: the pink esthetic score. Clin Oral Implants Res. 2005, 16:639-44. 10.1111/j.1600-0501.2005.01193.x [9] Available via license: CC BY 4.0

Variables	0	1	2
Papilla (mesial)	Missing	Incomplete	Complete
Papilla (distal)	Missing	Incomplete	Complete
Tissue contours	Unnatural	Virtually natural	Natural
Gingival level	>2 mm	1-2 mm	<1 mm
Alveolar process	Clearly resorbed	Slightly resorbed	No difference
Coloring	Clearly difference	Slight difference	No difference
Texture	Clearly difference	Slight difference	No difference

Patients’ Aesthetic and Functional Satisfaction

**Figure 7:**
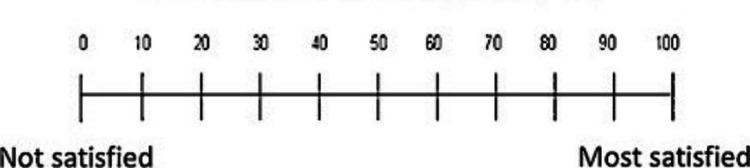
Visual Analog Scale

The Visual Analog Scale (VAS) was used by setting a scale of 0-100 and asking the patient to put the number expressing his degree of satisfaction with the treatment in the appropriate field on the form (Figure 7).

Statistical analysis

Data were collected and exported to Microsoft Excel 2013 (Microsoft Corporation). Then, statistical tests were conducted using Statistical Package for the Social Sciences (SPSS) version 26 (IBM SPSS Statistics, Armonk, NY, USA), with a significance level of 0.05. The Mann-Whitney U test was used to study the difference between the two study groups during each of the follow-up periods.

## Results

Sample description

The sample consisted of 20 abutments of 20 dental implants in 10 patients. Patients’ ages ranged between 19 and 62 years, with a mean of 41.5 years and a standard deviation (SD) of 16.8 years (Table 2).

**Table 2:** Descriptive statistics for the ages of the study patients

	Arithmetic mean	Standard deviation	Minimum	Maximum
Age	41.5	16.8	19	62

**Figure 8:**
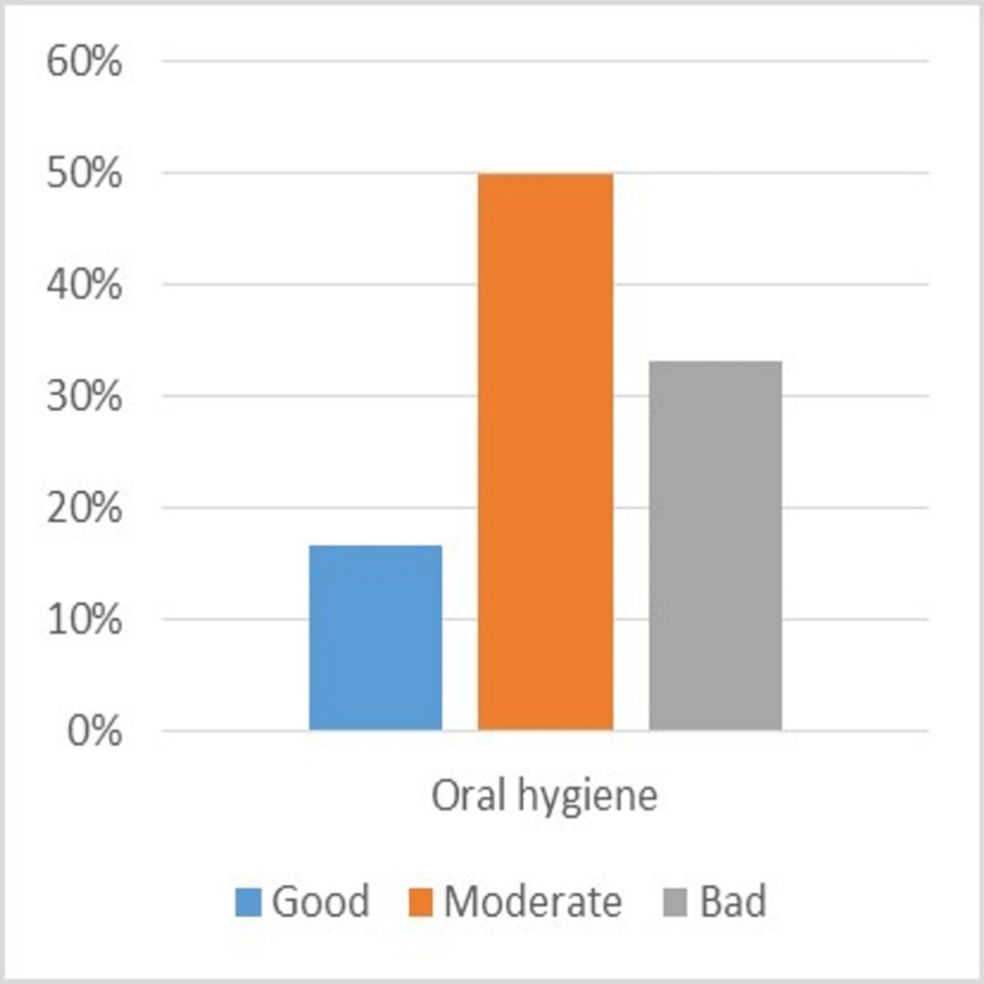
Sample characteristics (oral hygiene)

Good oral hygiene was observed in 16.7% of the patients, moderate in 50%, and poor in 33.3%. Moreover, none of the study patients were smokers (Figure 8).

Gingival biotype

**Figure 9:**
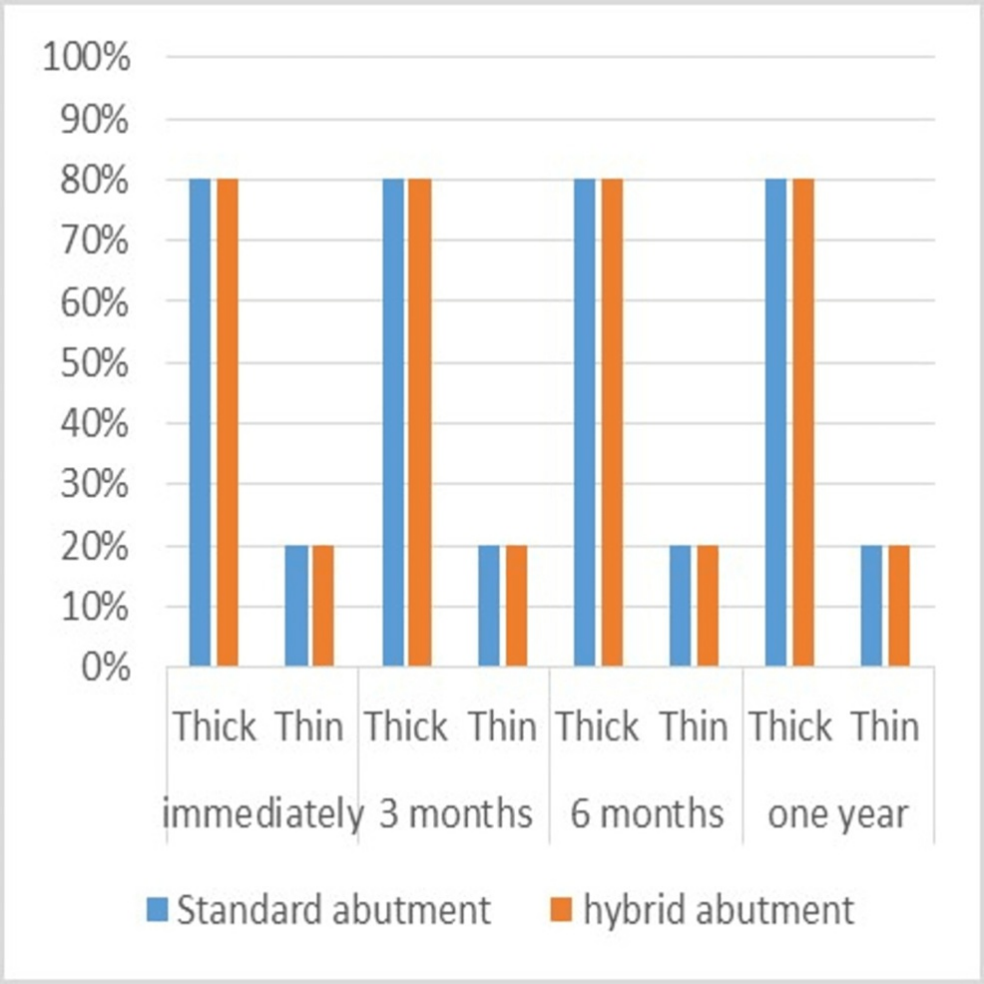
Percentages of gingival biotype assessment in the two study groups during the follow-up periods

The percentage of the thick biotype was 80%, and the percentage of the thin biotype was 20% in each of the two study groups during the follow-up periods (Table 3). Therefore, there were no significant differences between the two groups (Figure 9).

**Table 3:** Frequencies and percentages of gingival biotype assessment in the two study groups during the follow-up periods There are absolutely no differences between groups, so no statistical analysis is needed.

Follow-up after cementation	Gingival biotype	Standard abutment		Hybrid abutment	
		Frequency	%	Frequency	%
Immediately	Thick	8	80%	8	80%
	Thin	2	20%	2	20%
	Total	10	100%	10	100%
Three months	Thick	8	80%	8	80%
	Thin	2	20%	2	20%
	Total	10	100%	10	100%
Six months	Thick	8	80%	8	80%
	Thin	2	20%	2	20%
	Total	10	100%	10	100%
One year	Thick	8	80%	8	80%
	Thin	2	20%	2	20%
	Total	10	100%	10	100%

Interdental papilla filling

**Figure 10:**
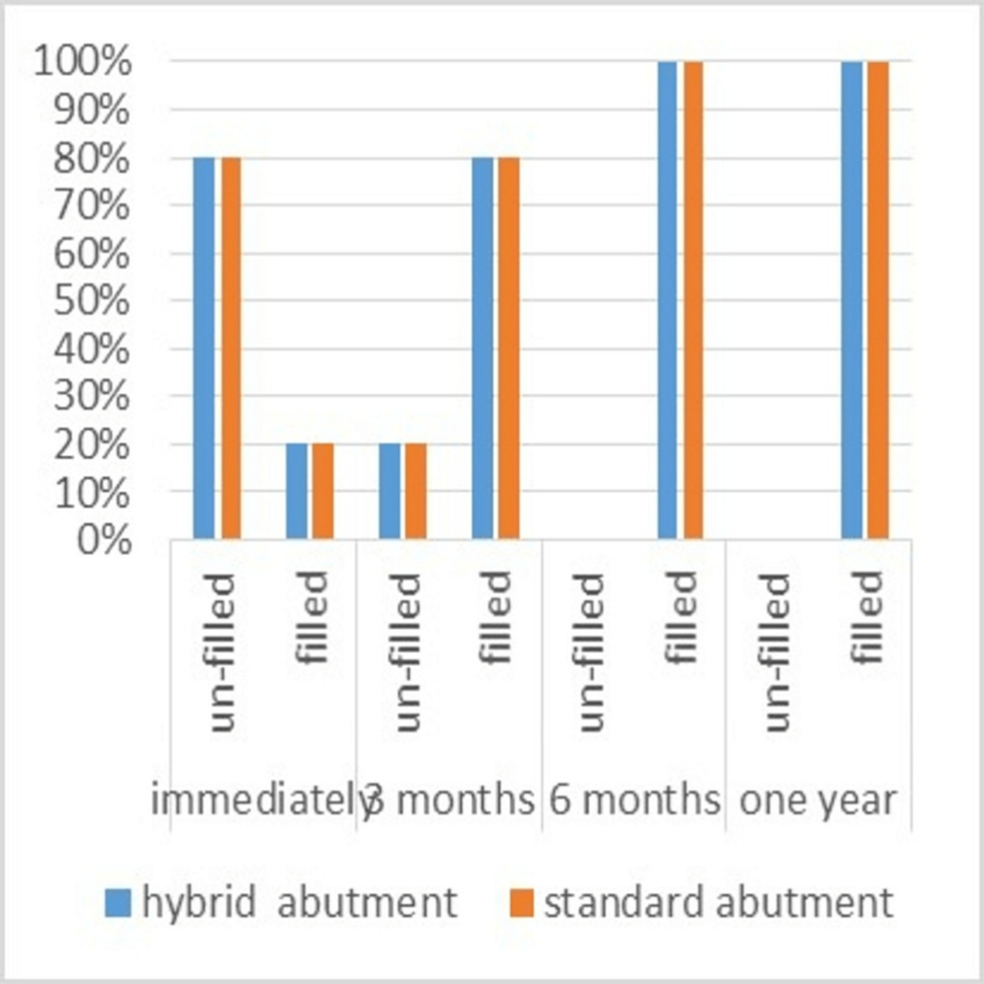
Percentages of papilla filling assessment in the two study groups during the follow-up periods

The percentage of filled papillae was 20%, while the percentage of unfilled papillae was 80% immediately after cementation in both groups. After three months, the percentage of filled papillae became 80% in both groups; however, after six months and one year, all interdental spaces were filled in all samples (Table 4) without any differences between groups (Figure 10). Furthermore, there were no differences in the papillae’s shape between the two groups.

**Table 4:** Frequencies and percentages of papilla filling assessment in the two study groups during the follow-up periods There are absolutely no differences between groups, so no statistical analysis is needed.

Follow-up after cementation	Papilla	Standard abutment		Hybrid abutment	
		Frequency	%	Frequency	%
Immediately	Unfilled	8	80%	8	80%
	Filled	2	20%	2	20%
	Total	10	100%	10	100%
Three months	Unfilled	2	20%	2	20%
	Filled	8	80%	8	80%
	Total	10	100%	10	100%
Six months	Unfilled	0	0%	0	0%
	Filled	10	100%	10	100%
	Total	10	100%	10	100%
One year	Unfilled	0	0%	0	0%
	Filled	10	100%	10	100%
	Total	10	100%	10	100%

Patients’ aesthetic satisfaction

Immediately and three months after cementation, patients’ evaluation was 8 in both groups, with no statistically significant differences between them (P = 0.684 and P = 0.529, respectively). Six months and one year after cementation, patients in both groups gave 9 as a rating, with no statistically significant differences between the two groups (P = 0.739 and P = 0.631, respectively) (Table 5).

**Table 5:** Effect of abutment type on patients’ aesthetic satisfaction during the follow-up periods The Mann-Whitney U test was used to detect significant differences in patients’ aesthetic satisfaction between the two groups. SD: standard deviation

Follow-up after cementation	Abutment type	Mean	SD	Minimum	Median	Maximum	P value
Immediately	Hybrid	7.8	0.4	7	8	8	0.684
	Standard	7.5	1.0	5	8	8	
Three months	Hybrid	8.2	0.4	8	8	9	0.529
	Standard	7.9	0.7	6	8	9	
Six months	Hybrid	8.6	0.7	7	9	9	0.739
	Standard	8.4	1.0	6	9	9	
One year	Hybrid	8.7	0.5	8	9	9	0.631
	Standard	8.3	1.3	5	9	9	

Patients’ functional satisfaction

Immediately and three months after cementation, patients’ evaluation was 8 in both groups, with no statistically significant differences between the two groups (P = 0.796 and P = 0.739, respectively). After six months, there were no statistically significant differences between the two groups with a median of 8 for standard abutments and 8.5 for hybrid abutments, while one year after cementation, patients in both groups had a median of 9, with no statistically significant differences between the two groups (P = 0.684) (Table 6).

**Table 6:** Effect of abutment type on patients’ functional satisfaction during the follow-up periods The Mann-Whitney U test was used to detect significant differences in patients’ functional satisfaction between the two groups. SD: standard deviation

Follow-up after cementation	Abutment type	Mean	SD	Minimum	Median	Maximum	P value
Immediately	Hybrid	7.6	1.3	6	8	10	0.796
	Standard	7.3	1.8	4	8	10	
Three months	Hybrid	8.3	0.7	8	8	10	0.739
	Standard	7.9	1.1	5	8	9	
Six months	Hybrid	8.6	0.7	8	8.5	10	0.631
	Standard	8.2	1.3	5	8	10	
One year	Hybrid	8.9	0.6	8	9	10	0.684
	Standard	8.7	0.8	7	9	10	

## Discussion

Various treatment options for replacing extracted teeth were used, starting with removable prosthodontics and ending with dental implants. A dental implant is one of the therapeutic options that restore functional and aesthetic aspects, in addition to its role in improving the patient’s psychological state [10]. Both removable and fixed implant-supported prostheses improve the patient’s quality of life compared to conventional prostheses [11]. The intimate connection between the implant and its abutment is an essential factor for the long-term success and stability of prostheses over implants [12].

Abutments vary in their shapes, uses, and methods of manufacture. Standard abutment is the most common type, for its advantages such as low cost and gaging with the implant body [5], in addition to the possibility of preparing it inside or outside the mouth. However, it also has some problems such as misalignment of the finish line with the gingival line, and its metallic color may appear under full ceramic prosthesis; moreover, it is difficult to secure the right angulation for this abutment in all cases.

The hybrid abutment consisting of a metal base with a ceramic cover of lithium disilicate provides proper angulation for the prosthesis’ placement, and it has the advantages of both titanium and ceramics, including improved aesthetics, perfect biocompatibility, and high mechanical properties [7].

Therefore, this study aimed to conduct a clinical comparison between standard and hybrid abutments in terms of the state of peri-implant gingival tissues and patients’ aesthetic and functional satisfaction (immediately, three months, six months, and one year) after the final prosthesis cementation.

The G-Power software was used to calculate the sample size. The sample consisted of 20 abutments of 20 implants in 10 patients, divided into two equal groups: the first group included 10 implants (AnyRidge®, MegaGen, Seoul, South Korea) with 10 standard abutments of the company, while the second group included 10 implants with 10 hybrid abutments (ZrGen, MegaGen, Seoul, South Korea), as each patient received two implants either adjacent or symmetrical in the same jaw, in order to standardize the oral conditions at each patient.

When the recovery period after implantation was over, the prosthetic procedures were started, as the impression was taken in the closed tray technique using an alternative to the laboratory implant and the appropriate transfer, which has flat sides and undercut areas to allow reorientation in the impression after removing from the mouth [13]. The closed tray technique has been adopted as it is preferred when implants are placed in the posterior region of the jaw.

The gingival mask was injected similarly to placing the gingiva inside the mouth. Then, the impression was poured using phosphate-bonded gypsum powder (Maruvest Speed, Megadental, Büdingen, Germany).

Additional silicon was also used for its high accuracy in copying details, in addition to being the best impression material in terms of dimensional stability, as the percentage of the dimensional change does not exceed 0.06% during the first 24 hours after taking the impression, while the percentage was 0.1% for polyether and 0.5 % for condensation silicon [14,15]. Both standard and hybrid abutments were placed over the alternative to the laboratory implant to ensure the placement of the abutment over the implants and their relationship with the rest of the teeth. The required modifications were made to the abutments to reach the appropriate position to receive the final prostheses.

Clinical evaluation of the peri-implant gingival tissues was carried out to determine whether its biotype was thick or thin. We found that the percentage of the thick biotype was 80% and that the percentage of the thin biotype was 20% in each of the two study groups during the follow-up periods. Therefore, there were no significant differences between the two groups. These results agreed with the results of the study of Lops et al., which was conducted on 15 patients (five thin biotype and 10 thick biotype). They reported no differences between the standard titanium abutment and the hybrid abutment in terms of the effect on the gingival biotype and color change after fixing the final prosthetics [16]. Another study stated that there was no difference in the color change of the gingiva, whether they were of the thin or thick biotype and whatever type of final abutment was used [17].

In contrast, the study by Martínez-Rus et al. showed a decrease in the degree of the color change of the gingiva by increasing its thickness when using a standard titanium abutment [18].

Implant position and the connection type between the abutment, the implant, and the cortical bone around the implant will determine whether there will be sufficient support for the peri-implant tissue, so the implant must be placed within the bone appropriately from all directions (medial, lateral, vestibular, and lingual) [19].

Tarnow et al. found that as the distance between the top of the alveolar bone and the contact point is 3-5 mm, the gingival papillae occupy the space by 98%-100%, and as this distance is 6 mm, the percentage is 56%, while as it is 7 mm, the percentage is 27%. Moreover, the percentage decreases to only 10% when this distance is 8 mm [20].

For the papilla filling evaluation, our study found that there were no differences in the percentage of the filled papilla and its shape between the two study groups. Thus, we agreed with the study that evaluated the percentage of filling of papillae based on PES when using a standard or hybrid abutment in an implant to restore a single tooth and found the same results [21].

To be able to assess patients’ aesthetic and functional satisfaction, the Visual Analog Scale has been approved as the easiest for patients and the most widely used in studies evaluating patient satisfaction with therapeutic procedures in general [22,23]. Each patient was asked to fill out the form himself to ensure that there was no bias.

This study showed no significant differences between the two groups in terms of patients’ aesthetic satisfaction. With regard to the standard abutment, these results agreed with another study, which stated that all patients were satisfied with the aesthetic results of using the titanium standard abutment without any change in the color of the soft tissues surrounding the implant during one year of follow-up [24].

The results of the current study also agreed with the results of a systematic review that stated that there was no difference in patient satisfaction with aesthetic aspects, whether the abutment used was standard titanium or hybrid, or fully zirconia [25]. In contrast, a new study mentioned that patients reported higher aesthetic results after using the hybrid abutment compared to the standard abutment despite using the same assessment index (VAS) [26].

This study showed that the evaluation of patients’ functional satisfaction in both groups was equal, and therefore, there were no statistically significant differences between the two groups during the follow-up periods. Regarding the hybrid abutment, these results agreed with the results of the recent study that showed a percentage of 100% for patient satisfaction with functional aspects and clinical performance when using this abutment. This study used the patient satisfaction scale graded from 1 to 4 [27]. Furthermore, our study correlated with the results of another study, which reported a percentage of 97.5% for patient satisfaction in terms of functional aspects. Functional problems only appeared in two of 25 cases; in the first case, the hybrid abutment was separated from the implant, and in the second case, the ceramic part was separated from the metal base [28], as the ​​contact area between the metal part and the ceramic part is the weakest point of the hybrid abutment and plays an important role in the long-term clinical success [29].

Naturally, the present study has its limits. The first one is the limited number of patients enrolled. The second limitation is that they were restored with single crowns and not with more complex restorations such as partial fixed prostheses.

## Conclusions

We found that standard and hybrid abutments can be used in single-tooth implants without significant differences between them in terms of affecting the peri-implant gingival tissue and patients’ aesthetic and functional satisfaction. However, hybrid abutment requires longer and more complicated laboratory procedures, so we recommended using standard abutment over the single-tooth implant to reduce work steps and resulting problems.
